# Diagnosis by metagenomic next-generation sequencing of a *Talaromyces marneffei* bloodstream infection in an HIV-negative child: A case report

**DOI:** 10.3389/fped.2022.903617

**Published:** 2022-08-15

**Authors:** Aimei Yang, Yan Hu, Peiling Chen, Guilang Zheng, Xuejiao Hu, Jingwen Zhang, Jing Wang, Chun Wang, Zijian Huang, Yuxin Zhang, Yuxiong Guo

**Affiliations:** ^1^Department of Pediatric Intensive Care Unit, Guangdong Provincial People's Hospital, Guangdong Academy of Medical Sciences, Guangzhou, China; ^2^Department of Laboratory Medicine, Guangdong Provincial People's Hospital, Guangdong Academy of Medical Sciences, Guangzhou, China; ^3^Department of Pediatrics, Guangdong Provincial People's Hospital, Guangdong Academy of Medical Sciences, Guangzhou, China

**Keywords:** *Talaromyces marneffei*, bloodstream infection, HIV negative, metagenomic next-generation sequencing, case report

## Abstract

**Background:**

*Talaromyces marneffei* (TM) bloodstream infections are life- threatening in immunocompromised individuals. The lack of specific clinical features for these infections and poor sensitivity associated with routine examination procedures make diagnosis challenging. Untimely diagnosis and delayed antifungal treatment threatens the life of such patients.

**Case description:**

We report a case of a TM bloodstream infection, confirmed by the results of blood culture, of a child who was HIV negative and possessed a *CD40LG* gene mutation. A diagnosis of TM was established by blood metagenomic next-generation sequencing (mNGS) of the patient's blood, which was confirmed by microbiological culture of blood. On admission, this previously healthy male patient was 8-months of age, who presented with recurrent fever and a cough of 6-days in duration. His condition did not improve after antibacterial treatment for 5-days, with significant and recurrent fever and worsening spirit. He was referred to the Department of Pediatrics in our tertiary medical institution with a white blood cell count of 21.5^*^10∧9/L, C-reactive protein of 47.98 mg/L, and procalcitonin of 0.28 ng/mL. A bloodstream infection was not excluded and blood was collected for microbial culture. The patient received a 1-day treatment of cefoperazone sulbactam and 6-days of imipenem cilastatin. Symptoms did not improve and fever persisted. Blood was submitted for mNGS analysis and within 14-h, 14,352 TM reads were detected with a relative abundance of 98.09%. Antibiotic treatment was immediately changed to intravenous amphotericin B combined with oral itraconazole. The condition of the child gradually improved. Blood culture showed TM on the 7th day after hospitalization, confirming bloodstream infection. After the 13th day of hospital admission, the patient's body temperature dropped close to 38°C and was discharged on the 30th day of hospitalization. Oral itraconazole was prescribed with follow up at the outpatient clinic.

**Conclusions:**

HIV-negative patients with *CD40LG* mutations may be potential hosts for TM. TM infections are rare in children and their detection by conventional microbial culture methods are inadequate for an early diagnosis. mNGS is a rapid detection method that permits early diagnosis of uncommon infectious agents, such as TM, allowing for improved patient outcomes.

## Introduction

The temperature-dependent dimorphic fungus, Talaromyces marneffei (TM), is epidemic in tropical Asia especially in; Thailand, northeastern India, Vietnam, Laos, Malaysia, Myanmar, Cambodia, Taiwan, Hong Kong, and Mainland China (1–3). This emerging fungal disease occurs in healthy individuals but is more common in immunocompromised or immunosuppressed individuals. Because of the extremely high mortality rate, TM-infections are a serious medical issue. Herein, we report rapid diagnosis of a child's TM-bloodstream infection (TM-BSI) by metagenomic next-generation sequencing (mNGS). Diagnosis was confirmed by blood culture identification of the fungus. The patient received combined therapy of amphotericin B and itraconazole. With treatment, the child's clinical symptoms significantly improved with normal body temperature upon hospital discharge.

## Case description

An 8-month-old Chinese boy was referred to this tertiary hospital on 29 December, 2021 for fever and cough, 6-days in duration. On admission vital signs were body temperature 37.3°C, blood pressure 82/54 mmHg, pulse 132 beats per minute, respiratory rate 32 breaths per minute, and poor spirit. The superficial lymph nodes were palpable in the neck and behind the ear, a few moist rales were heard over both lungs. The child's abdomen was soft, liver and spleen were not palpable, and the neurological examination was normal. With only a fever, the child was sent to a local hospital for blood tests. Results of those test were white blood cell (WBC) 24.62^*^10∧9/L, neutrophil percentage (N%) 44.1%, and C-reactive protein (CRP) 32.4 mg/L. Antibacterial treatment for 5-days produced no improvement and the child was referred again to our hospital. The parents indicated that the patient had normal psychomotor development and physical growth, but had recurrent episodes of respiratory infections from delivery until now.

Upon admission, routine blood test results were WBC 21.5^*^10∧9/L, N% 40.6%,CRP 47.98 mg/L, procalcitonin (PCT) 0.28 ng/ml, (1, 3)-β-D-glucan test (G test), purified protein derivative (PPD) test, and T-spot test for tuberculosis were negative. Serological tests for HIV, cytomegalovirus (CMV), herpes virus, and rubella virus were negative. A chest computed tomography (CT) scan showed lung inflammation and a small amount of liquid in the right pleural cavity, foliar consolidation in the upper left lung, and multiple calcified nodules in bilateral axillary fossa, with multiple enlarged lymph nodes in the bilateral supraclavicular fossa. Echocardiographic showed no dilation of the coronary arteries. Brain magnetic resonance imaging (MRI) and abdominal ultrasound results were normal. Lymphocyte counts, as measured by flow cytometry, were CD3^+^ cells (total T lymphocytes) 58.57% (peripheral blood reference range 49.1–83.6%); CD19^+^ cells (total B lymphocytes) 35.4% (higher than normal 6.5–27.0%); CD3^+^CD4^+^ T helper cells (Th) 50.06% (28.2–62.8%); CD3^+^CD8^+^ T cells (Ts) 6.8% (10.2–40.1%); Th/Ts 7.36% (0.7–2.8%); and NK (natural killer) cells 4.23% (7–40%). The IgG, IgA, and IgM concentrations were; 0.49 g/L (reference 2.32–14.11 g/L), 0.07 g/L (reference 0.0–0.83 g/L), and 0.94 g/L (reference 0.0–1.45 g/L), respectively. The patient received antibacterial (cefoperazone sulbactam, imipenem cilastatin) treatment and supportive care, including human gamma globulin. The child's condition did not improve. Temperature exceeded 39°C, with WBC 16.5^*^10∧9/L, N% 52.0%, and CRP 62.38 mg/L.

**Figure 1:**
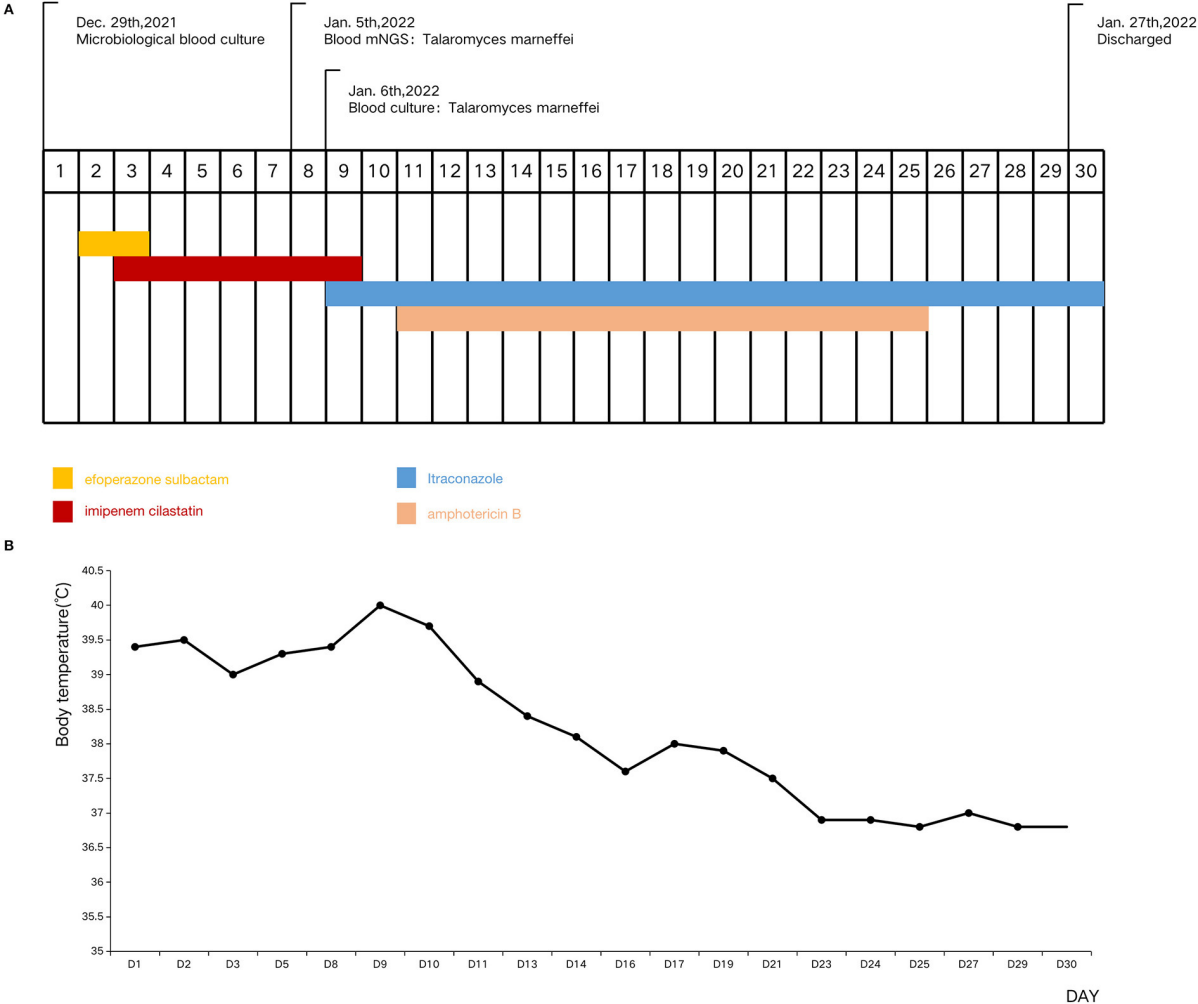
Timeline of the patient's clinical manifestations and treatment. **(A)** Timeline of the patient's tests and treatment. **(B)** Timeline of the patient's body temperature.

**Figure 2:**
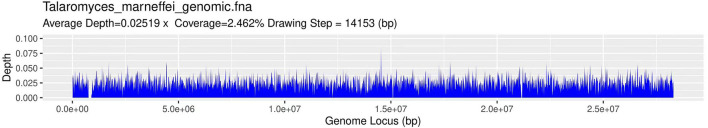
The coverage depth for*Talaromyces marneffei*.

After 8-days of administration and a poor response to antibiotic therapy, blood was collected for mNGS (Figure 1). Fourteen hours later TM was identified by mNGS. Microbiological blood culture confirmed TM infection on the 9th day of hospitalization, and with the quick mNGS results (Figure 2), intravenous amphotericin B (0.1–0.5 mg/kg/day, with gradual increase every 2 days) and oral itraconazole (4 mg/kg/dose bid) were administered. The patient's condition improved dramatically. On the 13th day of hospitalization, the patient's fever was <38°C. He was discharged from the hospital on December 30, 2022 when his body temperature had remained normal for 1 week. This was the 30th day of hospitalization.

**Figure 3:**
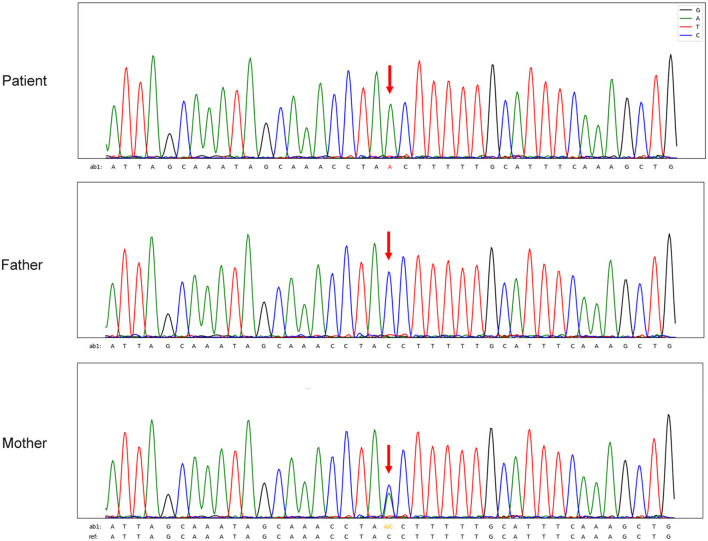
Sequencing maps of *CD40LG* gene variants in the infant and his parents.

The patient was young and HIV- negative. However, his lymphocyte counts and immunoglobulin levels were abnormal and therefore congenital immunodeficiency was not excluded. Blood exon sequencing was performed to identify possible CD40LG gene variations. Analysis demonstrated the proband to be a hemizygous intron variant (c.346+1G>T) of the CD40LG gene (NM_000074), which was inherited from his mother, who was heterozygous. The father had the wild-type sequence (4) (Figure 3). The patient was diagnosed with TM-BSI and a CD40LG mutation.

For follow up, the patient has returned to the hospital for outpatient examination. His general condition remains normal without any apparent abnormalities. Oral itraconazole treatment has continued.

## Discussion

Fungal bloodstream infections are a life-threatening condition with global mortality rates surging more than 5-fold in the past decade (5). Overall, TM infection rates range from 4–14%, with the mortality rate of TM infection 10–30%. Those who do not receive antifungal therapy have a >50% mortality rate (6–8). Clinical case studies of TM-BSI of children are rare. Some studies have shown the mortality rate to be 16.7–17.2% despite the use of antifungal therapy and supportive care (9, 10).

TM infections in HIV-negative individuals have recently increased, due to increased numbers of immunocompromised individuals (8). Such patients typically have differing degrees of immunosuppression due to; primary immunodeficiency (PIDs), hematological malignancies, transplant rejection, and administration of corticosteroids or immunosuppressive agents (11). TM infections can disseminate into many organs, with a rapid progressive course that can be life-threatening, without timely antifungal therapy.

The clinical manifestations of TM infection are non-specific, with the severity of disease dependent upon the degree of host immune-suppression (12). Clinical features of HIV-negative patients with TM infection include; recurrent fever, cough, hepatosplenomegaly, lymphadenopathy, weight loss, and gastrointestinal abnormalities (3). TM infections have various imaging manifestations including; pulmonary nodules and voids, patchy shadows, mediastinal lymph node enlargement, pleural effusion, liver and spleen enlargement, as well as bone destruction. Patients can be misdiagnosed with other pathogenic infectious agents. Qiu et al. (13) found that 38.1% (24/63) of TM patients were misdiagnosed with tuberculosis and 7.9% (5/63) were misdiagnosed with bacterial pneumonia. Our patient presented with a history of repeated and prolonged fever. Examination prompted lung inflammation and pleural effusion, with multiple swollen lymph nodes. However, after several days of antibiotic treatment, the condition had not improved or even worsened. This made us to consider infection by a special pathogen, such as TM.

Microbiological cultures from a variety of sites such as blood, bone marrow, respiratory, liver, cerebrospinal fluid, urine, and stool are the primary method for diagnosis of TM (12), but is time consuming and has a limited positivity rate. It is worth noting that TM is ta pathogen identified based on its characteristic morphologic and dimorphic properties, with a mycelial growth form at 25°C and a yeast form at 37°C (14). Conventional microbiological cultures are often more prone to false-negatives. There are some tests for the diagnosis of TM such as; enzyme-linked immunosorbent assay (ELISA), nested polymerase chain reaction (PCR) and real-time PCR, and Mp1p antigen-detecting enzyme immunoassay (EIA), but these require a predefined range of suspicious pathogens (15–17). Laboratory fungal diagnosis relies on isolation of the pathogen and/or direct microscopic examination that are neither sensitive nor specific, with fungal identification often very difficult (18). Further, most antigen/antibody detection methods [e.g., (1, 3)-β-D-glucan and galactomannan antigens] are broadly non-specific and insensitive detecting components of a number of different fungi (19). Therefore, these situations do not adequately provide for an early diagnosis, nor provide for adequate clinical guidance we should adopt more effective diagnostic methods, blood-mNGS was used in this case.

mNGS is a new and preferred method for detection of this and other rare pathogens. mNGS identification of systemic fungal infection provides the possibility for rapid infectious agent diagnosis (20). Since it sequences all DNA present, mNGS is a useful tool for identification of those fungi that are difficult to culture or at very low cell number. Nonetheless, the mNGS method does have a few restrictions; colonization differentiation, infection and contamination, interference of human nucleotide sequences (DNA), matched reads, and cell-free DNA extraction (21, 22). TM infections are not common in HIV-negative patients, hence evaluation by mNGS permits proper identification.

For this case, the local hospital provided empirical, anti-bacterial treatment by administration of meloxicillin-sulbactam and cefoperazone-sulbactam.Imipenem cilastatin was administered at our hospital for 1-week. With recurrent and persistent high fever, a non-bacterial infection was suspected. Blood mNGS confirmed TM as the causative agent, since there are no therapeutic guidelines for TM infections in children, amphotericin B and itraconazole are recommended as initial antifungal treatments (23). The targeted therapy improved the patient's condition significantly. The pathogen was identified as TM, an opportunistic fungus that is relatively uncommon clinically and mainly occurs in patients with immunodeficiency. The parents also indicated that the child had multiple respiratory infections after birth. We considered the possibility of an immunodeficiency disease and genetic tests were performed. Genetic analysis of this child found a CD40LG gene mutation, which is related to Hype-IgM syndromes, characterized by normal or elevated levels of polyclonal serum IgM and low levels of serum IgG, IgA and IgE (24). The exact basis for patient susceptible to TM due to immunodeficiency caused by the CD40LG mutation is unknown. In our case, the IgG levels of the child decreased, which was consistent with changes in immunoglobulins common to children infected with TM (25). In addition, we also found a lower percentage of NK cells in the patient, which may be due to increased activation-induced cell death and decreased production of NK cells through defective CD40L-CD40 interaction (24).

In summary, TM infections in HIV-negative individuals present in a non-specific manner with complicated clinical manifestations and an unclear infectious route. If the infected person fails to receive timely antifungal treatment, prognosis is often poor, with the possibility of a life threatening situation. Therefore, it is essential to obtain a TM diagnosis as early as possible. mNGS may permit rapid diagnosis of TM infection. In addition, when TM infections occur in HIV-negative individuals, clinicians should be alert to the possibility of immunodeficiency, with relevant genetic test completed as soon as possible.

## Conclusions

HIV-negative patients with CD40LG mutations may be potential hosts for TM. TM infections have a high mortality rate in HIV-negative children. To insure early diagnosis and timely treatment, it is necessary to be familiar with TM clinical manifestations and effective diagnostic procedures. This case report demonstrates the value of mNGS for the diagnosis of blood fungal infection, as a diagnostic tool useful for rare pathogens. Timely and effective therapy will improve patient prognosis and will save lives.

## Data availability statement

The original contributions presented in the study are included in the article/supplementary materials, further inquiries can be directed to the corresponding author/s.

## Ethics statement

The studies involving human participants were reviewed and approved by the Medical Ethics Committee of the Guangdong Provincial People's Hospital, China. Written informed consent to participate in this study was provided by the participants' legal guardian/next of kin.

## Author contributions

AY, YG, and YZ contributed to conception and design of the study. AY wrote the first draft of the manuscript. YH, GZ, JZ, JW, CW, and ZH were responsible for the manuscript data collection and literature review. PC completed the follow-up. XH provided mNGS data analysis. All authors revise, read, and review submissions. All authors contributed to the article and approved the submitted version.

## Conflict of interest

The authors declare that the research was conducted in the absence of any commercial or financial relationships that could be construed as a potential conflict of interest.

## Publisher's note

All claims expressed in this article are solely those of the authors and do not necessarily represent those of their affiliated organizations, or those of the publisher, the editors and the reviewers. Any product that may be evaluated in this article, or claim that may be made by its manufacturer, is not guaranteed or endorsed by the publisher.
